# MiR-205 Dysregulations in Breast Cancer: The Complexity and Opportunities

**DOI:** 10.3390/ncrna5040053

**Published:** 2019-11-19

**Authors:** Yajuan Xiao, Brock Humphries, Chengfeng Yang, Zhishan Wang

**Affiliations:** 1 Department of Toxicology and Cancer Biology, University of Kentucky, Lexington, KY 40536, USA; yajuan.xiao@uky.edu (Y.X.); chengfeng.yang@uky.edu (C.Y.); 2 Cancer Center, Integrated Hospital of Traditional Chinese Medicine, Southern Medical University, Guangzhou 510315, China; 3 Center for Molecular Imaging, Department of Radiology, University of Michigan, Ann Arbor, MI 48109, USA; brhu@med.umich.edu

**Keywords:** miR-205-5p (miR-205), breast cancer, metastatic breast cancer, triple negative breast cancer, luminal A/B breast cancer, Her2^+^ breast cancer

## Abstract

MicroRNAs (miRNAs) are endogenous non-coding small RNAs that downregulate target gene expression by imperfect base-pairing with the 3′ untranslated regions (3′UTRs) of target gene mRNAs. MiRNAs play important roles in regulating cancer cell proliferation, stemness maintenance, tumorigenesis, cancer metastasis, and cancer therapeutic resistance. While studies have shown that dysregulation of miRNA-205-5p (miR-205) expression is controversial in different types of human cancers, it is generally observed that miR-205-5p expression level is downregulated in breast cancer and that miR-205-5p exhibits a tumor suppressive function in breast cancer. This review focuses on the role of miR-205-5p dysregulation in different subtypes of breast cancer, with discussions on the effects of miR-205-5p on breast cancer cell proliferation, epithelial–mesenchymal transition (EMT), metastasis, stemness and therapy-resistance, as well as genetic and epigenetic mechanisms that regulate miR-205-5p expression in breast cancer. In addition, the potential diagnostic and therapeutic value of miR-205-5p in breast cancer is also discussed. A comprehensive list of validated miR-205-5p direct targets is presented. It is concluded that miR-205-5p is an important tumor suppressive miRNA capable of inhibiting the growth and metastasis of human breast cancer, especially triple negative breast cancer. MiR-205-5p might be both a potential diagnostic biomarker and a therapeutic target for metastatic breast cancer.

## 1. Introduction

Breast cancer is posing a tremendous threat to women’s health globally, and is the most prevalent female malignancy in the world [1]. Traditionally, breast cancer has been classified as in situ (ductal and lobular) or as an invasive disease based upon morphological markers [2]. Advancements in technology has allowed breast cancer to be further classified into different subtypes based upon molecular markers defined by immunohistochemistry (IHC): Estrogen (ER)/progesterone (PR), human epidermal growth factor receptor 2 (HER2), or triple negative breast cancer [2]. More recently, proteomics and gene-expression profiling of breast cancer has allowed for subtyping of breast cancer based upon its molecular profile. The most commonly used classification system stratifies breast cancer into four subtypes: Luminal A, luminal B, HER2 positive (+), and basal-like breast cancer [3]. Because the treatment of breast cancer has evolved from surgery to include systemic therapy determined by different molecular mechanisms and clinical phases, breast cancer therapy has achieved tremendous progress in patient survival [4]. However, despite this advancement, breast cancer remains the leading cause of cancer death in women, with 535,000 deaths in 2016 in 195 countries or territories across the world [5]. Approximately 90% of breast cancer-related death is ascribed to breast cancer metastasis [6]. Metastatic breast cancer is an incurable disease and has an unfavorable prognosis, with an average 5-year survival rate of around 25% [7]. Our limited understanding of the mechanisms underlying breast cancer metastasis constrains the current efficacy of therapy. This is especially true for triple negative breast cancer (TNBC), which is defined as estrogen receptor (ER), progesterone receptor (PR), and HER2 negative. TNBC usually displays higher recurrence, more aggressive metastasis, and worse clinical outcome compared with other breast cancer subtypes. Although TNBC shares many clinical characteristics, TNBC tumors display a higher level of molecular heterogeneity compared to other subtypes. Therefore, understanding the underlying mechanisms of TNBC is imperative, and more novel therapeutic strategies based on molecular mechanisms of TNBC need to be developed to improve patient prognosis [8].

MicroRNAs are endogenous small (19–25 bases) non-coding single-stranded RNAs nucleotides, which repress their target genes by typically pairing to the 3′UTR of mRNAs. In animals, mature microRNAs are derived from long primary transcripts (pri-miRNAs). Pri-miRNA is transcribed by RNA polymerase II from short open reading frames of DNA which do not encode for any proteins [9]. After being transcribed in the nucleus, pri-miRNA is cleaved by nuclear RNAse III Drosha, resulting in stem–loop intermediates termed precursor miRNA (pre-miRNA) [10,11]. After this cleavage event, pre-miRNA is exported out of the nucleus and subsequently truncated by another RNase in the cytoplasm, cytoplasmic RNase III Dicer, forming a 22 nt double-stranded RNA [12,13,14]. Afterwards, the strand with a less stable 5′ end is degraded, while the remaining strand is referred to as a mature miRNA [15,16]. The primary function of miRNAs is to downregulate expression of their target genes. They accomplish this by varying mechanisms, which include RNA degradation, induced decapping, induced deadenylation, altered cap protein binding, reduced ribosome occupancy, and sequestration of mRNA [17]. Usually, the seed sequence of miRNAs paired by base with 3′UTRs of mRNAs. The seed sequence usually consists of 2–8 bases and starts at the second and ends at the eighth nucleotide region counted from the 5′ end of miRNAs [18,19]. The seed sequence plays a significant role in recognition of target mRNAs and provides an important basis for miRNA target prediction.

MiRNAs are involved in the epigenetic regulation of cancer development. The functions of different miRNAs vary in different cancers, but can generally be classified as oncogenic or tumor suppressive. It has been reported that miRNAs participate in cancer initiation, tumorigenesis, proliferation, metastasis, epithelial mesenchymal transition (EMT), stemness maintenance, and therapeutic resistance by downregulating target oncogenes or tumor suppressive genes. Therefore, these dysregulated miRNAs in cancers are found as biomarkers for diagnosis and potential targets for cancer treatment [20].

**Figure 1:**
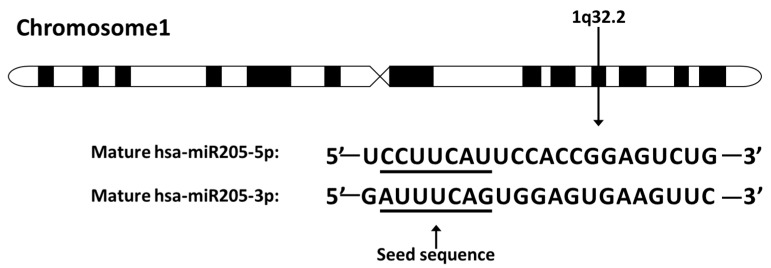
The miR-205 location on chromosome and sequence. MiR-205 locates on human chromosome 1q32.2. The seed sequences of miR-205-5p and miR-205-3p are underlined. The miR-205 discussed in this review refers to miR-205-5p.

In humans, miR-205 is located on chromosome 1q32.2 and is composed of a highly conserved structure (Figure 1). More specifically, miR-205 sits between the second and the third exon of *LOC642587*. MiR-205 is normally expressed in the breast, prostate, and thymus of humans and regulates development of these organs [21]. However, in cancer, miR-205-5p expression is context- and cancer-specific. For example, miR-205-5p is suppressed or silenced in breast, prostate, melanoma, and renal cell carcinoma, but is overexpressed in non-small cell lung carcinoma, bladder cancer, ovarian cancer, endometrioid adenocarcinoma, head and neck cancer, and esophageal adenocarcinoma [22,23,24,25,26,27,28,29].

**Figure 2:**
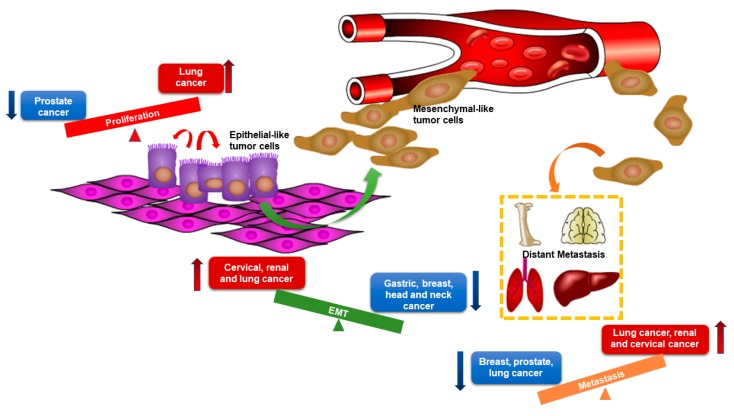
The distinct roles of miR-205-5p in different types of cancers. Red arrows represent the facilitating effects of miR-205-5p and blue arrows represent the suppressive effects of miR-205-5p. The graph shows opposite roles of miR-205-5p in tumor proliferation, epithelial–mesenchymal transition (EMT), and metastasis among different types of cancers. MiR-205-5p exerts promoting effects in cancers listed in red boxes and exerts suppressive effects in cancers listed in blue boxes.

Since microRNAs have multiple targets, thus elucidating their underlying effects on cancers is complex. In support of this, miR-205-5p has been shown to play multiple roles in different cancers (Figure 2). For example, miR-205-5p is involved in embryogenesis, especially in epithelium morphogenesis [30], because it promotes epithelium differentiation in endoderm and ectoderm [31], but miR-205-5p is also involved in epithelial maintenance [32]. Furthermore, in gastric, breast, head, and neck cancer cell lines, epithelial identities, including morphology, increased E-cadherin expression, decreased Vimentin and N-cadherin expression, are restored by re-expressing miR-205-5p [33,34]. However, upregulation of miR-205-5p in cervical, lung, and renal cancer cell lines under hypoxic conditions promotes EMT by targeting ASPP2, one of the downstream apoptosis-stimulating proteins of p53 [35]. Interestingly, miR-205-5p was shown to suppress metastasis by downregulating LPR1, a migration factor, in lung cancer, which indicates a context-dependent function of miR-205-5p, even in the same type of cancer [36]. In addition to converging data with cell differentiation and migration among cancer types, miR-205-5p is also identified as both a positive and negative regulator of proliferation. In prostate cancer, miR-205-5p was shown to arrest cell growth by repressing the mitogen-activated protein kinase (MAPK) and androgen receptor (AR) pathways [37,38]. Whereas an inverse effect was reported in lung cancer as miR-205-5p inhibited PTEN signaling to enhance proliferation [39]. This review aims to summarize the expression and function of miR-205-5p in breast cancer subtypes and highlights the differential roles and targets of miR-205-5p in breast cancer initiation and progression.

## 2. MiR-205-5p Expression in Normal Breast Tissues and Its Dysregulation in Different Subtypes of Breast Cancer Tissues

**Figure 3:**
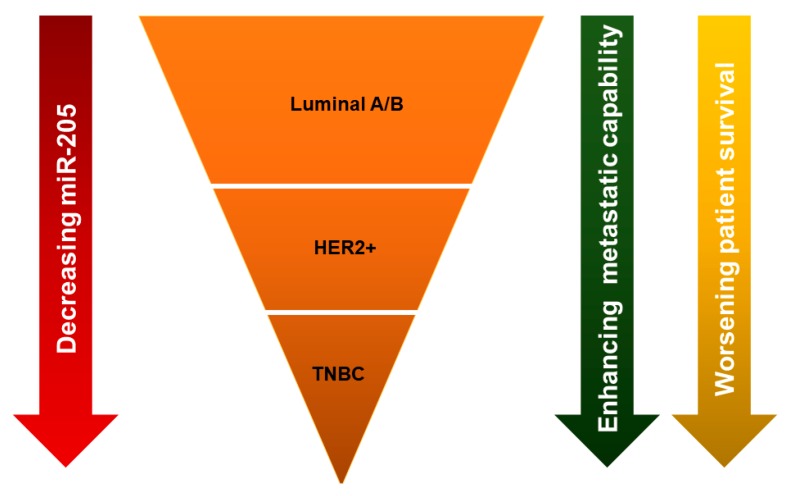
Differential expression levels of miR-205 among breast cancer subtypes. The expression of miR-205-5p is lower in HER2^+^ than luminal A/B, and triple negative breast cancer (TNBC) has the lowest miR-205-5p level compared with the other subtypes. Decreasing miR-205-5p expression level is associated with enhanced metastatic capability and worsening of patient survival.

Originally, miR-205-5p expression was observed exclusively within myoepithelial cells in lobules and ducts of normal breast tissue [32]. However, compared to normal breast tissue, expression of miR-205-5p is decreased in breast cancer [40]. It is interesting to note that, although miR-205-5p expression is lost in breast cancer, the relative levels of miR-205-5p loss varies between subtypes. The level at which miR-205-5p is lost correlates with a variety of mechanisms for their biological behavior (Figure 3). MiR-205-5p is upregulated in ER/PR^+^ breast cancer compared with HER2^+^ breast cancer, and shows a significant correlation with ER/PR status [41]. Among all subtypes of breast cancer, triple negative breast cancers (TNBCs) express the least miR-205-5p [32,40,42]. Additionally, metastatic breast cancers express lower levels of miR-205-5p than non-metastatic breast cancers [32,43]. In support of this, our previous study found that lower expression of miR-205-5p was associated with worse recurrence-free survival and distant metastasis-free survival [44]. Low levels of miR-205-5p in serum has also been reported as a diagnostic biomarker for breast cancer patients, as well as an unfavorable clinical prognostic factor [45].

## 3. Mechanisms of MiR-205-5p Expression Regulation

Since the alteration of miR-205-5p expression is involved in breast cancer development, it is necessary to understand the regulatory mechanisms of miR-205-5p. MiR-205-5p is an intragenic miRNA, which is located within the intron of its host gene, as mentioned previously. One mechanism of miR-205-5p regulation is methylation of CpG islands within its promoter. In breast epithelial cells, overexpression of ERBB2 can drive miR-205-5p promotor methylation via Ras/Raf/MEK/ERK pathway-mediated DNMT upregulation [46]. In TNBC cells, Piovan et al. found that p53 binds a responsive element of the miR-205-5p host gene to increase miR-205-5p expression [47]. It was concluded that the high frequency of TP53 mutations in basal-like breast cancer correlated with suppressive expression of miR-205-5p [47]. Hairy and enhancer of split-1 (HES1) response elements were also found within the miR-205-5p promoter, and exert a negative effect on miR-205-5p expression in breast cancer [48].

## 4. Breast Cancer Subtype-Specific Roles of MiR-205-5p Dysregulation and Underlying Mechanisms

### 4.1. MiR-205-5p Dysregulation and Function in Hormonal Receptor Positive (Luminal A and B Subtypes) Breast Cancer

Hormonal receptor positive breast cancer constitutes approximately 80% of breast cancer [49]. Targeting the ER/PR-driven pathway has achieved great success in patients with hormonal receptor positive breast cancer. Tamoxifen, as a first-line endocrine therapy for ER/PR^+^ breast cancers, binds to the estrogen receptor to competitively block estrogen-induced target gene expression, which leads to suppression of cancer cell proliferation [50]. However, up to 30% of ER^+^ breast cancer patients are initially resistant to tamoxifen, and approximately 40% of patients with ER^+^ breast cancer that initially responded to tamoxifen eventually develop resistance [51]. A previous study demonstrated that miR-205-5p is involved in tamoxifen resistance of hormonal receptor positive breast cancer [52]. The molecular mechanisms of tamoxifen resistance can be summarized as follows: (1) Increased bidirectional ER/growth factor (GF) receptor cross-talk; (2) activated ER signaling downstream kinases, such as ERK, MAPK, and AKT; (3) absence of HDAC recruitment to ER corepressors; (4) acetylation of EGFR promotes receptor tyrosine phosphorylation and activation; and (5) corepressor of ER complexes inactivated and coactivator complexes activated [51]. Zhang et al. found that long noncoding RNA-ROR (lncRNA-ROR) was involved in the molecular mechanism of tamoxifen resistance, and found that lncRNA-ROR induced resistance by silencing miR-205-5p [52]. They generated a tamoxifen-resistant MCF7/TR5 cell line by chronic low-dose tamoxifen treatment [52], and found that the level of lncRNA-ROR was raised and miR-205-5p was decreased in these cells [52]. This negative correlation between lncRNA-ROR and miR-205-5p was further analyzed by *Renilla* luciferase activity, which indicated that lncRNA-ROR acted as a sponge and downregulated miR-205-5p expression [52]. Although they did not fully uncover how miR-205-5p is involved in tamoxifen resistance, it could be speculated that miR-205-5p might play a role in activating a downstream ER-dependent kinase.

Besides its function in endocrine therapy resistance, miR-205-5p has also been found to participate in cell proliferation of ER/PR^+^ breast cancer. In normal tissue, mammary duct cells are organized asymmetrically to an apical pole toward surrounding tissue and a basal pole that interfaces with the stroma and vasculature, and are mediated by tight junctions [53]. Dysfunction of the asymmetrical growth of the mammary duct is one of the mechanisms of breast cancer progress. Angiomotin (AMOT) is an adaptor protein that regulates tight junctions, and thus the spatial distribution of apical polarity proteins which controls apical asymmetry. Studies have shown that AMOT activates the ERK1/2 pathway to drive cell proliferation in ER^+^ breast cancer [54], and that miR-205-5p inhibits cell growth by direct targeting of AMOT in MCF-7 breast cancer cells [55]. This suggests that miR-205-5p function is critical for regulating breast cancer growth.

### 4.2. MiR-205-5p Dysregulation and Function in Her2-Enriched (HER2^+^) Breast Cancer

HER2-enriched (HER2^+^) breast cancer is a distinct subtype characterized as high expression of HER2-regulated genes and low expression of hormonal receptors [56,57,58]. HER2^+^ is driven by the overexpression of ERBB2 (HER2), an oncogene coding for a tyrosine kinase receptor belonging to the human epidermal growth factor receptor (EGFR) family [59,60,61]. The other three members of the EGFR family are HER1 (EGFR), HER3 (ERBB3), and HER4 (ERBB4). After being bound by ligand, the receptors dimerize to either homo- or heterodimers to activate several intracellular signaling pathways, such as the Ras/MAPK and PI3K/Akt, which ultimately promote proliferation, survival, and motility [62]. The HER2/HER3 heterodimer in particular plays a significant role in breast cancer proliferation, and HER3 was found to be frequently co-expressed with HER2 [63]. Furthermore, even in the absence of ligand binding HER2, activation of the PI3K/Akt survival pathway strongly depends on HER3 phosphorylation [64]. Recent studies have shown that miR-205-5p directly targets HER3. This results in the inhibition of proliferation in SKBr3, MCF7, and MDA-MB-231 breast cancer cell lines [40,42].

Until the development of trastuzumab, a HER2-specific recombinant humanized monoclonal antibody, the diagnosis of HER2^+^ breast cancer had a poor prognosis [60,65,66]. Although trastuzumab has achieved great success in the targeted therapy of HER2^+^ breast cancer, the recent emerging trastuzumab-acquired resistance of cancer cells provides yet another barrier to overcome [67]. Work looking into the development of resistance has elucidated complex answers: Some classified HER2^+^ tumors express low HER2 expression, partial masking of the HER2 epitope, and/or poor HER2–T-DM1 complex internalization, among others [68]. In addition to acquired resistance, cancer stem cells (CSCs) also play an important role in trastuzumab resistance [69]. CSCs characteristically have an inherent drug resistance, and therefore are likely the cause of tumor recurrence. Overexpression of miR-205-5p in breast cancer stem cells contributed to the development of trastuzumab resistance by lowering ERB2 and EGFR expression [69]. Furthermore, De Cola et al. indicated that miR-205-5p was significantly upregulated in HER2^+^ patient-derived breast cancer stem cells (BCSCs) compared with the same cells grown in differentiating spheroid conditions, sphere-derived adherent cells (SDACs) [69]. Knocking-down the expression of miR-205-5p in BCSCs upregulated HER2 and EGFR and sensitivity to Lapatinib. Mechanistically, this group determined that p63 is a direct target of miR-205-5p, and there is a feedback loop between p63 and miR-205-5p, which determines some of the features of BCSCs [69].

### 4.3. MiR-205-5p Dysregulation and Function in Triple Negative Breast Cancer

Triple negative breast cancer (TNBC) is a group of breast cancer subtypes characterized by traits of aggressive tumor proliferation, early distant metastasis, and enhanced cancer relapse. Previous studies have identified miR-205-5p as a critical regulator of these three characteristics by modulating different signaling pathways.

Dysregulation of the cell cycle is one of the hallmarks of proliferative dysfunction. In addition to identifying putative response elements in the promoter of miR-205-5p, Piovan and colleagues also analyzed the effects of miR-205-5p on cell cycle progression [47]. They found that growth inhibition of MDA-MB-231 and BT-549 overexpressed with miR-205-5p was not due to changes in apoptosis, but rather that a reduction in E2F1 expression, a direct target of miR-205-5p, impaired G1/S phase transition, and promoted cell senescence [47]. Additionally, suppressing miR-205-5p contributed to tumor growth, likely by driving E2F expression and accelerating the G1/S transition [47]. In contrast, a separate study demonstrated that in normal mammary myoepithelial and stem cells, miR-205-5p enhanced cell growth by regulating the cell cycle through phosphatase and tensin homolog (PTEN), a well-studied tumor suppressor gene [70]. Increasing miR-205-5p resulted in reduced PTEN expression, which was found to promote cell G1/S transition with more cells in S phase and fewer cells in G0 phase [71].

The cancer microenvironment is critical to progression, because it not only directly interacts with cancer cells, but also extensively affects the biological behavior of the cell, including tumor proliferation. LAMC1 is a member of the laminin super family, a component of the extracellular matrix (ECM) and a significant ECM regulator [72]. In MDA-MB-231 TNBC cells, LAMC1 was found to be downregulated by miR-205-5p and was confirmed as a direct target [47]. Both repressing LAMC1 by shRNA and overexpression of miR-205-5p inhibited clone formation ability in MDA-MB-231 cells [47]. Although LAMC1 has multiple effects on biological activities, including cell adhesion, proliferation, migration, and differentiation, this study at least demonstrated that miR-205-5p inhibits breast cancer growth partly by targeting LAMC1 [47,72].

Alterations in EGFR signaling pathways drive tumor initiation and progression. As with HER2^+^ breast cancer, miR-205-5p can regulate TNBC growth by modulating members of the EGFR signaling pathway. Typically, tumors are unable to grow beyond a volume of 1–2 mm^3^ due to the lack of nutrients perfusing the whole tumor [73]. Therefore, cancer survival and growth relies upon angiogenesis, or the creation of new blood vessels. The high-mobility group box (HMGB) family is a family of non-histone DNA-binding proteins that participate in the formation of new blood vessels by binding to receptors, such as advanced glycation end products (RAGE), to improve angiogenic cytokine release [73]. In breast cancer patients, one member of this family (HMGB3) was found to be overexpressed, and its expression correlated with worse prognosis [49]. Elgamal et al. discovered that overexpression of miR-205-5p in MDA-MB-231 and BT549 TNBC cell lines had similar proliferation rates to those that suppressed HMGB by RNAi [49]. They also demonstrated that HMGB3 is the direct target of miR-205-5p by a dual-luciferase assay, thus proving that miR-205-5p impeded breast cancer growth by targeting HMGB3 [49].

In a different study, 16 miRNA expression profiles were scanned across 32 TNBC samples and their corresponding non-tumor adjacent tissues [74]. In these samples, the miR-205-5p levels were significantly suppressed in the lymph node metastatic group [74]. Compared with their non-tumor adjacent tissues, expression of miR-205-5p was found be decreased more than 2-fold in tumor tissues [74]. These data suggest that miR-205-5p may play a significant role in TNBC metastasis.

Epithelial–mesenchymal transition (EMT) has been demonstrated as a major contributor to cancer metastasis and invasion [75,76]. Polarized epithelial cells undergo a biochemical transformation into cells that display a more mesenchymal-like phenotype and morphology with enhanced migratory capacity, invasiveness, resistance to apoptosis, and productivity of ECM [77]. Through EMT, cancer cells acquire the critical features needed to initiate aggressive invasion, which advances to subsequent metastatic cascade [78]. On the molecular level, EMT is concordant with the upregulation of factors implicated in cytoskeletal changes and the mesenchymal phenotype, including ZEB1, ZEB2, SNAIL, SLUG, TWIST, SMAD 2/3, Vimentin and N-Cadherin, and repressed markers implicated in the maintenance of an epithelial phenotype, including E-Cadherin, Occludin, Claudin, and Laminin [79]. One of the first studies on miR-205-5p observed that expression of miR-205-5p and the miR-200 family in breast cancer was upregulated in epithelial-like phenotype compared with mesenchymal-like phenotype. They also negatively correlated this change in miR-205-5p expression with the expression level of ZEB1 and SIP1, and positively correlated with the expression level of E-cadherin. Then, they demonstrated that miR-205-5p and the miR-200 family targeted ZEB1 and SIP1 to regulate EMT/MET by restoring expression of E-cadherin in the kidney epithelial cell line [34]. Lee et al. further confirmed that miR-205-5p directly targeted ZEB1 and ZEB2 in MDA-MB-231 TNBC cells by a luciferase reporter assay [80]. They also found that polycomb group protein Mel-18, a well-known regulator of chromatin modifications [81], upregulates miR-205-5p expression by methylation of a CpG island within its promoter [80]. More mechanistically, they also found that Mel-18 prevented DNMT recruitment to the promoter of miR-205-5p to reduce methylation effects and rescue miR-205-5p expression [80]. The miR-205-5p re-expression inhibited the invasive and migratory phenotype of MDA-MB-231 and MCF-7 cells driven by shRNA knockdown of Mel-18 (shMel-18) [80]. Furthermore, in vivo NOD/SCID mice models were also established by MCF-7 cells treated with a control shRNA (shcon) or shMel-18. They found more aggressive and mesenchymal-like tumors in mice injected with knockdown of Mel-18, and an upregulation of mesenchymal markers and downregulation of epithelial markers [80]. In addition to being involved in angiogenesis and cell proliferation, HMGB3 is another target of miR-205-5p that is responsible for EMT in breast cancer [82]. In a transwell invasion assay, miR-205-5p was shown to suppress MDA-MB-231 and BT549 invasion [82]. These data suggest that miR-205-5p is an important regulator of EMT, and those phenotypic processes that are associated with EMT, such as cell migration.

Integrins are a family of heterodimeric transmembrane receptors that bind extracellular matrix proteins, which are extensively involved in tumor cell adhesion, migration, invasion, and metastasis [83,84]. Integrin α5 (ITGA5) is one of the members of integrins and is found to play an essential role in breast cancer metastasis and growth [85,86]. According to our recent study, we found that ITGA5 expression was increased upon suppression of miR-205-5p in TNBC [44]. Overexpressing miR-205-5p in MDA-231-LM2 and SUM-159 TNBC cell lines drastically inhibited their migration and invasion capabilities [44]. Through rescue experiments, we found that re-expression of ITGA5 restores those metastatic characteristics. Further probing revealed that miR-205-5p directly targeted, and thus downregulated, ITGA5 and suppressed TNBC metastasis through the Src/Vav2/Rac1 signaling pathway [44].

Angiogenesis is not only necessary for tumor survival, but also plays an essential role in the dissemination and establishment of tumor metastases. In order to escape the primary tumor, tumor cells hijack endothelial cells to form new blood vessels. These neovessels allow for tumor cells to enter into the bloodstream. Therefore, the more highly vascularized the tumor is, the greater the chance that a cancer cell can metastasize [87]. Not surprisingly, miR-205-5p is also involved in angiogenesis. Wu et al. confirmed miR-205-5p paired to the 3′UTR of VEGF-A by a luciferase reporter assay and hypothesized that the observed reduced lung metastasis of MDA-MB-231 cell line transfected with miR-205-5p in vivo was partly due to repressed VEGF-A expression [40]. Cancer-associated fibroblasts (CAFs), a critical tumor stroma participant for tumor angiogenesis, were also found to be regulated by miR-205-5p in breast cancer. MiR-205-5p targets YAP1 (Yes-associated protein) in normal fibroblasts (NFs), preventing CAFs transformation from NFs, and suppresses angiogenesis, invasion, and metastasis of breast cancer cells in vivo [88].

Cancer stem cells mediate cancer relapse, metastasis, and therapy resistance. The stemness of cancer cells encompasses the capacity of asymmetrical self-renewal to one stem cell and one progenitor cell, as well as symmetrical division into two identical stem cells which can retain the original capacity for self-renewal [89]. MicroRNAs are a significant regulator of CSCs by targeting some critical members of pathways that regulate CSC phenotypes or EMT, including Bmi1, Suz12, Sox2, Klf4, and ZEB1/2 [34,90,91,92]. Sempere et al. reported that the low expression level of miR-205-5p was associated with increased relapse rate in patients with triple negative breast cancer that were enriched with breast cancer stem cells, implicating miR-205-5p in cell stemness [32]. In regard to EMT, the loss of E-cadherin during EMT discharges β-catenin from the plasma membrane and activates canonical Wnt signaling by β-catenin translocation to the nucleus, which is an essential pathway for cancer stem cells [93,94,95]. Loss of epithelial polarity also induces the Hippo pathway to promote mammosphere formation, an assay that can define stemness in vitro [96,97,98]. Because, disturbed epithelial polarity contributes to self-renewing symmetric divisions of CSCs that drives those CSC subgroups surging among cancer cell pools [99]. To further uncover the relation between miR-205-5p and self-renewing symmetric division, Chao et al. found that ZEB1, a direct target of miR-205-5p, and suppression of miR-205-5p results in enhanced ZEB1 expression. Enhanced ZEB1 expression drove self-renewing symmetric division and maintenance of stemness of CSCs by redistribution of NUMB, a protein primarily participating in controlling the polarity of stem cells [48]. Meanwhile, overexpression of miR-205-5p in MDA-MB-231 and BT-549 TNBC cell lines inhibited NOTCH2 expression, another direct target of miR-205-5p, which resulted in a loss of stem cell identity [48]. Intriguingly, Notch2 and its ligand, Jagged-1, could also upregulate HES1 expression to inhibit miR-205-5p, and constituted a negative feedback loop of Notch2/miR-205-5p/ZEB1 signaling in breast cancer cells [48].

The CSC-like property suppressive effects by miR-205-5p were also observed in our previous study [44]. A well-established serum-free suspension culture mammary sphere formation assay was performed to assess the CSC-like property of SUM-159 TNBC cells [44]. We found that the counts of spheres formed were reduced by miR-205-5p expression and increased by ITGA5 re-expression. These data support our in vivo data of nude mouse orthotopic mammary xenograft models, showing that miR-205-5p acts as a CSC inhibitor [44].

## 5. Potential Diagnostic and Therapeutic Values of MiR-205-5p in Breast Cancer

### 5.1. MiR-205-5p Abnormal Expression as a Potential Diagnostic Marker for Breast Cancer

Different types of cancers have varying microRNA molecular profiles. Therefore, characterizing specific microRNA levels for a particular cancer is helpful not only for confirming the diagnosis, but also for treatment decision, staging, and prognosis. Therefore, understanding the relationship between microRNAs and the cancer and subtype will contribute to a much more accurate diagnostic, subtype, grading, response, and prognosis prediction tool. In regards to miR-205-5p, Berber found that a 5-fold decrease in miR-205-5p expression levels compared to normal breast tissue was accurate to predict breast cancer lymph node metastasis [74]. This paper was able to obtain a sensitivity of 68.8% and specificity of 81.3% in a sample size of 32 patients with TNBC who underwent radical mastectomy and axillary dissection [74]. This study demonstrates the usefulness of miRNAs, particularly miR-205-5p, in patient prognosis prediction, and revealed that miR-205-5p expression levels might be one of the candidate indicators for TNBC metastasis [74].

It is well-known that intracellular miRNAs are excreted by cells into the blood stream after being packaged into exosomes or microvesicles [100]. This suggests that circulating miRNAs serve as ideal biomarkers for earlier detection of cancer [101]. Shaker and colleagues collected blood samples from 100 breast cancer patients and 30 healthy females, and identified the expression levels of miRNAs [102]. In this case, miR-205-5p was identified as a critical differential circulating miRNA between these groups. The sensitivity of miR-205-5p for detection of breast cancer was 98.8% and the specificity was 100% by using the optimal cutoff value [102]. Zhang et al. also performed a case control study on 58 breast cancer patients and 93 healthy controls [72]. The diagnostic proficiency of serum miR-205-5p for breast cancer was achieved at a sensitivity of 86.2% and a specificity of 82.8% [72]. Together, these studies demonstrate that miRNAs, and more specifically miR-205-5p, are potent diagnostic and prognostic prediction tools.

### 5.2. The Role of MiR-205-5p in Breast Cancer Treatment: A Potential Therapeutic Agent and Regulation of Drug Response/Resistance

As microRNAs are shown to be tremendous tumor suppressive effects, the prospects of anti-microRNAs and microRNAs themselves as therapeutics are promising. Since miR-205-5p shows differential effects between breast cancer subtypes, miR-205-5p therapy likely has the most patient benefit in TNBC. This is because many studies have shown that miR-205-5p is highly downregulated in TNBC, and re-expression of miR-205-5p in TNBC consistently inhibits tumor growth, stemness, and metastasis. However, in order to use miRNAs in the clinic, many barriers revolving around preventing microRNA degradation in vivo, inefficient systemic and targeted delivery, and reduced uptake by cells remains to be solved [103].

MicroRNAs are extensively involved in therapeutic resistance [20,104,105,106]. The luminal A subtype of breast cancer is less sensitive to chemotherapy than other subtypes. Instead, treatment of luminal subtypes of breast cancer consists of endocrine therapy, which is highly effective. Although endocrine therapy is effective, chemotherapy is still required to be administered to those with early stage, or with high risk and advanced disease states. In addition to their involvement in drug resistance, miRNAs can also sensitize cells to chemotherapy, resulting in enhanced response and better patient prognosis. For example, circulating miR-205-5p was identified as a potential predictor of resistance to chemotherapy of epirubicin plus paclitaxel in luminal A subtype [107]. Additionally, miR-205-5p was also found to sensitize MDA-MB-231 TNBC and MCF-7 cells to docetaxel, both in vitro and in vivo [108]. In another study, Hu et al. analyzed 30 breast cancer tissues from patients and found that miR-205-5p had a positive correlation with neoadjuvant chemotherapy response rate [109]. MCF-7/A02 and CALDOX are chemoresistant breast cancer cell lines derived from the chemosensitive cell lines MCF-7 and Cal51 cells, respectively. MiR-205-5p was found to be downregulated in MCF-7/A02 and CALDOX compared with their parental cell lines [109]. Re-expression of miR-205-5p re-sensitized MCF-7/A02 and CALDOX response to doxorubicin and docetaxol [109]. It was further revealed that over-expression of miR-205-5p blocked the PI3K/AKT pathway by targeting VEGF-A and FGF2, which resulted in increased apoptosis upon chemotherapy treatment [109]. MiR-205-5p was also suggested to be an important negative mediator of radiotherapeutic resistance [110]. Ionizing radiation (IR) enhanced hypermethylation of CpG islands of the host gene of miR-205-5p and suppressed miR-205-5p expression [110]. A miR-205-5p mimic attenuated the IR induced Bcl-w increase, malignancy, and lung metastasis in H460 and MDA-MB-231 cells [110].

## 6. Summary and Perspectives

**Figure 4:**
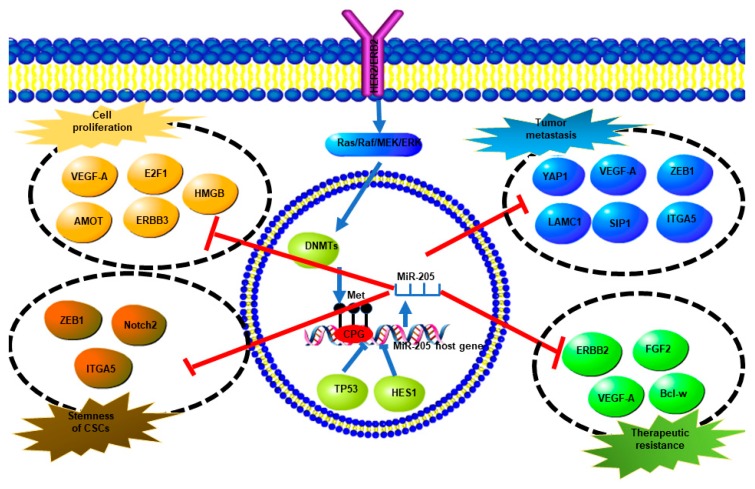
A summary of miR-205-5p expression regulation and direct targets of miR-205-5p and their biological effects. Overexpression of ERBB2 promotes methylation of the miR-205-5p promoter via the Ras/Raf/MEK/ERK pathway which upregulates DNMTs, which finally results in miR-205-5p downregulation. TP53 and HES also inhibit miR-205-5p expression. MiR-205-5p targets different genes directly to regulate cell proliferation, tumor metastasis, stemness, and therapeutic resistance.

The studies on miR-205-5p have shed light on the complicated molecular mechanisms of breast cancer initiation and development. In general, miR-205-5p plays a tumor suppressive role in breast cancer. It inhibits tumor growth, metastasis, EMT, cancer stem cell maintenance, and drug resistance (Figure 4). The miR-205-5p expression is reduced in breast cancer compared with normal breast tissue. The expression of miR-205-5p in breast cancer varies among subtypes, with hormonal receptor positive (ER^+^) expressing the highest levels, HER2-enriched expressing lower levels, and triple negative breast cancer expressing the lowest levels of miR-205-5p. In hormonal receptor positive breast cancer, miR-205-5p not only suppresses tumor growth, but also participates in tamoxifen sensitivity maintenance. In HER2-enriched breast cancer, miR-205-5p plays a controversial role, which on the one hand downregulates HER3 to inhibit HER2/HER3 dimerization and effects on tumor growth, and on the other hand, downregulates HER2 expression, thus inducing lapatinib resistance. In TNBC, miR-205-5p is more extensively involved in tumor growth, metastasis, EMT, and stemness maintenance. The known direct targets of miR-205-5p in breast cancer are summarized in Table 1.

Since more and more targets for miR-205-5p in breast cancer are being discovered, the mechanisms of how miR-205-5p regulate breast cancer development are expanding and are better understood. The roles of those target genes in breast cancer have been further discussed as well. Furthermore, the significance of miR-205-5p as a potential therapeutic target has been highlighted, as well as its target genes. However, there are still many questions that need to be answered in the future. Both miR-205-5p and the miR-200 family function similarly in breast cancer and even share some target genes, such as ZEB1 and SIP1 [34,74,115]. This opens up the possibility that these miRNAs share more commonalities, such as their regulating mechanism, as well as if they interact with each other, and even if these miRNAs have synergistic effects. The combination therapy of the two is also an understudied topic in the field. MiR-205-5p also interacts with other non-coding RNAs, such as lincRNA-ROR and lncRNA-PNUTS, which has opened a new horizon in studying miR-205-5p [52,116,117]. In addition, while studies clearly show that miR-205-5p displays strong tumor suppressive effects and has the potential to be a therapeutic agent for treating triple negative breast cancer, the challenge is how to safely and efficiently deliver miR-205-5p to tumor tissues.

## Figures and Tables

**Table 1:** A summary of the validated direct targets of miR-205-5p in breast cancer.

Direct Targets	Function of the Targets in Breast Cancer	Reference
AMOT	Regulator of spatial distribution of mammary duct epithelial cells	[55]
ERBB3	One of the EGFR family, co-function with ERB2 in activating the PI3K/Akt survival pathway	[40,42,111]
VEGF-A	Regulator of angiogenesis of tumors	[40,109]
HMGB	Nonhistone DNA-binding protein, participates in angiogenesis	[82,112]
YAP1	A transcription regulator of the Hippo signaling pathway	[88]
E2F1	Promoter of G1/S transition	[47]
PTEN	Suppressor of G1/S transition in mammary myoepithelial cells	[70]
LAMC1	A component of the extracellular matrix, regulating cancer microenvironment	[47]
ZEB1/ZEB2/SIP1	Regulating EMT	[34,80,113]
ITGA5	A member of integrin family, regulating tumor cell adhesion, migration, invasion, and metastasis	[44]
Notch2	Regulating cancer cell stemness	[48]
FGF2	Regulating cell survival	[109]
ERBB2	Activating p63 to maintain sensitivity to Lapatinib	[69,114]
Bcl-w	Mediating acquired IR-induced malignancy	[110]
